# Patient-appraised beneficial moments during inpatient psychiatric treatment

**DOI:** 10.1186/s12913-020-05617-4

**Published:** 2020-08-10

**Authors:** Cosima Locher, Ramin Mansour, Helen Koechlin, Stefan Büchi

**Affiliations:** 1 School of Psychology, Plymouth University, Plymouth, UK; 2 Clinic for Psychotherapy and Psychosomatics “Hohenegg”, Meilen, Switzerland; 3 Division of Clinical Psychology and Psychotherapy, Department of Psychology, University of Basel, Basel, Switzerland; 4 Department of Anesthesiology, Critical Care, and Pain Medicine, Boston Children’s Hospital, Harvard Medical School, Boston, MA USA

**Keywords:** Beneficial moments, Inpatients, Specific factors, Common factors, Qualitative, Content analysis

## Abstract

**Background:**

Psychiatric inpatients receive a multidisciplinary treatment approach, covering psychiatry, nursing, occupational therapy, and psychology. Research findings reveal that the effectiveness of any treatment is associated with three types of factors: specific (e.g., treatment techniques), common (e.g., clinician-patient relationship, patients’ expectations) and extra-therapeutic. However, there is little published research on the factors and events which inpatients themselves consider to be beneficial (‘beneficial moments’).

**Methods:**

Inpatients (*N* = 107) of a psychiatric clinic completed a questionnaire to elicit their appraisal of beneficial moments. A qualitative content analysis was applied. The coding procedure was conducted independently by two authors.

**Results:**

Self-appraised beneficial moments were found in five areas: therapy-specific components (number of quotations, *N* = 204), positive relationships (*N* = 140), clinical setting and environment (*N* = 52), inpatients’ new insights (*N* = 36), and factors unrelated to either therapy or the clinic (*N* = 30). In total, 44% of the quotations were related to specific factors, 49% to common factors, and 7% to extra-therapeutic factors.

**Conclusions:**

Inpatients judge both specific and common factors as crucial for the therapeutic benefit they gain during their stay at the clinic. Our results differ from meta-analytical findings, where the impact of specific factors on symptom improvement has shown to be much smaller (i.e., 17%) than appraised by patients in our study (i.e., 44%). Our study underlines the importance of a patient-centred care approach as well as shared decision making and patient-clinician communication. For clinical practice, knowledge of inpatients’ perspectives on beneficial moments is crucial in order to reinforce precisely these therapeutic components.

## Background

Psychiatric inpatients participate in a wide array of multidisciplinary treatments: inpatient care usually involves psychiatry, nursing, occupational therapy, and psychology [1]. Determining the effectiveness of therapy for psychiatric inpatients is a challenging task, particularly because of the difficulty in conducting blinded randomized controlled trials (RCTs) in this setting [2]. Nevertheless, a recent meta-analysis found that psychological therapy in acute inpatient settings was associated with small-to-moderate improvements in psychotic symptoms as well as moderate-to-large improvements in depression and anxiety at the end of treatment [3]. This finding is in line with various meta-analyses published over the last years, reporting that the estimated effect size related to the absolute efficacy of psychotherapy falls within the range of .75 to .85 [4]. Notably, there is little variability in the absolute effect size of various psychological interventions. For example, effect sizes for short-term psychodynamic psychotherapy are large and similar to those reported for cognitive-behavioural therapy (CBT) in anxiety disorders [5]. Similarly, Paterson, Karatzias [3] found no evidence to favour one specific type of psychological therapy over another regarding symptom relief in acute inpatient settings.

However, how the effectiveness of a psychological intervention is achieved still needs to be clarified. Since the late 1940s, psychotherapy research aimed to uncover the effect of ‘specific factors’ (e.g., treatment techniques and methods such as exposure, free association, skills training) which rely on a specific psychotherapy theory (e.g., CBT). Beyond their specific components, different psychotherapy approaches typically share a variety of so-called ‘common factors’ [4], including the interpersonal clinician-patient relationship [6] patients’ expectations [7], as well as the provision of a convincing rationale [8]. There is a widespread agreement among psychotherapy researchers that these common factors are significant mediators of outcome [9–12]. Along similar lines, a meta-analysis evaluating the efficacy of non-directive supportive therapy (i.e., therapies helping patients to talk about their experiences without applying specific psychological techniques) in adult patients suffering from depression reported that common factors are responsible for about half (49.6%) of the symptom improvement (i.e., indirectly measured by the effects of non-directive supportive therapy compared with control groups), whereas extra-therapeutic factors are responsible for about 33.3% (i.e., the effects of control groups) and specific factors for about 17.1% (i.e., the effects of non-directive supportive therapy compared with other therapies) of the improvement [13]. The variety of common factors that are indispensable to practising psychotherapy effectively have been summarized in the contextual model [14, 15].

Beyond the theory-driven discussion of whether and how much specific, common and extra-therapeutic factors contribute to treatment effectiveness, qualitative examinations give an insight into what patients themselves find helpful for their symptom relief. Accordingly, a broad spectrum of themes has been examined in significant events research [16], which represents a specific approach to study the patient-defined important events in the therapy process [17]. Common topics include (out)patients’ descriptions of helpful events [18], significant change events [19], as well as components of the therapeutic relationship which are perceived as crucially important [20]. Outpatients’ perspectives support the assumption that common and relational factors contribute more to positive behaviour change at the end of treatment than specific factors [17, 21]. Similarly, in a study where patients were asked to complete an instrument weighing preferences for specific empirical support against preferences for common factors, patients were found to value a satisfactory and empathic therapeutic relationship more than the empirical support of the intervention [22].

In the context of psychiatric inpatient care, qualitative examinations typically focus on the satisfaction with services [23], inpatients’ experiences in the acute psychiatric clinic [24, 25], as well as various assessments of ‘responsiveness’ [26]. However, there is little published information on factors or events which patients themselves appraise as beneficial. There are several reasons why this is important. Firstly, it is crucial for patient-centred care, which is based on shared decision making and communication [27]. Secondly, in order to inform inpatients about how psychotherapy works (such as the importance of their own expectations and the therapeutic alliance) [28], it is especially important to be aware of the patient’s subjective viewpoint. Thirdly, for clinical practice, a deeper understanding of inpatients’ perspectives on beneficial moments is critical in order to reinforce precisely these components of therapy [29].

The aim of this study was therefore to explore inpatients’ experiences of beneficial moments during their stay at an inpatient psychiatric clinic. We decided to examine subjective experiences of a good treatment in clinical practice rather than focusing on the criteria of efficacy, effectiveness, or efficiency that are typically applied in quantitative studies. Furthermore, we investigated whether the reported beneficial moments were associated with specific or common therapeutic factors, or extra-therapeutic factors, associated with the multidisciplinary treatments delivered in the clinic.

## Methods

### Setting / sample

The data are from a single Swiss clinic with 70 beds, located in a rural setting, for acute psychiatric disturbances with special focus on treatment of affective disorder and adjustment disorder, but no patients with drug dependency or schizophrenia. The treatment is multimodal with a focus on drug treatment and on psychotherapy including individual treatment (120 min/w direct contact) as well as several group therapies. The individual treatment is conducted by medical specialists. Furthermore, mindfulness-based body therapies (Shiatsu, Feldenkrais, Qi-Gong, Mindfulness-Based Stress Reduction [MBSR]) as well as creative therapies (Art Therapy, Music Therapy, Occupational Therapy) are offered in individual as well as group settings. Nursing is provided on an individual basis and the interaction with the patient group is structured according to the concept of milieu therapy.

The sample was drawn from all inpatients admitted to the clinic between January 1 and May 31, 2016. There was no selection or exclusion made. Of the 176 eligible inpatients, 107 patients (61%) filled out the questionnaire and participated in the study. All of these patients had a minimum stay of 4 weeks at the psychiatric clinic and were in the final stage of their stay.

### Questionnaire

We decided to construct a questionnaire instead of a semi-structured qualitative interview due to logistical and time-related reasons. Since our aim was to use an explorative approach to examine inpatient’s experiences of beneficial moments, we wanted to receive feedback from as many patients as possible – which was feasible through a questionnaire. We developed the questionnaire ourselves, applying an internal consensus exercise. In meetings with medical specialists, nurses and alternative therapists, we agreed on the term ‘beneficial moments’ (i.e., in German: “*magische Momente*”) to explore events that are associated with a healing process during patients’ stay at the clinic. Similar terms have also been applied in psychotherapy research (e.g., [30, 31]).

The questionnaire measured inpatients’ self-appraised beneficial moments during their stay at the psychiatric clinic. An open question format was chosen. The use of open-ended questions in a questionnaire is suitable for first insights into a novel research field [32, 33]. The questionnaire was part of a broader questionnaire of the psychiatric clinic. In detail, patients were asked to qualitatively describe a maximum of three ‘beneficial moments’ that they experienced during their stay at the psychiatric clinic which were of particular importance to them. We assumed that patients would describe the ‘beneficial moments’ which were most salient to their experience at the clinic.

The questionnaire consisted of additional quantitative questions (i.e., Likert Scale from 1 to 6) that focused at the following constructs: relationship with the medical specialists, plausibility of the treatment, expertise of the medical specialists, and personal involvement in the treatment process. The results of these questions are not reported in this qualitative study: The quantitative questionnaires were collected for internal clinical purposes and made it possible to obtain an initial rough overview.

The data collection was completely anonymous and participation was voluntary. All patients were considered to have full control over the extent to which they wanted to participate. The second author (RM) was responsible for patient recruitment. Patients received the questionnaire at the end of their stay at the clinic. The completed questionnaire was brought by the patients to the last appointment with the treating medical specialist. The questionnaire was reviewed by the Local Ethics Committee, Zurich, Switzerland. The project did not fall within the scope of the Human Research Act (HRA) and no consent was required because no health-related data were assessed.

### Qualitative analyses

The data has been evaluated by applying the structuring content analysis developed by Mayring [34]. For data analysis, the software Atlas.ti (https://atlasti.com) was used. The structuring content analysis was chosen since it is suitable for summary, structure, evaluation and analysis of large datasets.

The summary is one of the elementary components of the qualitative content analysis. It focuses on the identification of people’s key messages by reducing them to major subjects. In detail, we classified identical and similar passages by topic and then combined similar factors into major categories. This allowed us to identify the main themes. Hence, we preserved the original content while producing an inductive summary at a higher level of abstraction. After identifying the main themes, we grouped them in accordance with the contextual model, i.e., whether they represent specific, common or extra-therapeutic factors in the treatment process [4].

Two of the authors (CL and RM) were involved in the analysis. The first author (CL) holds a PhD in clinical psychology and psychotherapy. The second author (RM) is a medical specialist in psychiatry and psychotherapy. The authors share research interests on the impact of common factors on intervention effects. Using constructive feedback loops during the analytic process, the authors worked on minimizing the potential dominance of either professional background on the study results. In a first step, we analysed the transcripts independently (with each analyst using his or her coding scheme) and identified the main themes and factors. In the next step, we compared the two coding schemes, debated disagreements, and reached consensus on one scheme.

## Results

### Sample characteristics

A total of 107 patients (68% women and 32% men) completed their inpatient treatment in the clinic during data collection and were evaluated for this study. Patients had a mean age of 54.4 years. The profile of respondents was similar to the clinic patients as a whole.

### General description of responses and major themes

The patients’ responses varied in form and length. Some provided lists of one-word responses, while others answered in whole sentences or narrative phrases. They included one to three factors per answer, and there were no missing responses to the question we were analysing. Occasionally, patients used punctuation or underlined words for emphasis.

Twenty-one factors were generated from the beneficial moments which respondents noted. Our data showed that patient-appraised beneficial moments could be described by five main themes:

1. Specific therapy components
2. Positive relationships
3. The setting and the environment of the clinic
4. Inpatients’ new insights
5. Factors unrelated to either therapy or the clinic

**Fig. 1:**
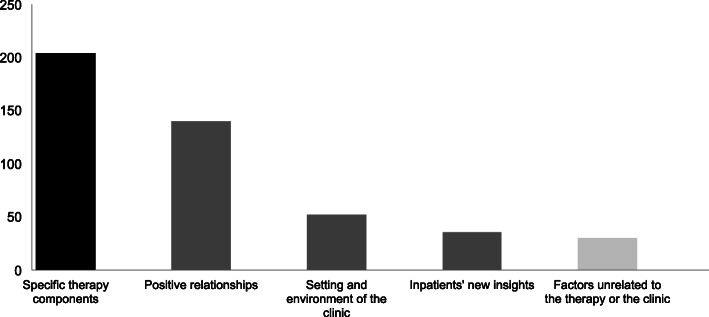
Number of Quotations for Main Themes. Note. Black bars are specific factors, grey bars are common factors and bright bars are extra-therapeutic factors

After identifying our main themes, we classified the factors in accordance with the contextual model, i.e., whether they represent specific, common or extra-therapeutic factors in the treatment process (see Fig. 1). Five factors could be associated with specific factors; 14 factors could be related to common factors. The remaining two were allocated to extra-therapeutic factors. Specific factors were mentioned 204 times (44% of the total number of quotations); common factors 228 times (49% of quotations); extra-therapeutic factors 30 times (7% of quotations).

In the following subsections, we provide more details about the factors which influenced patient-appraised beneficial moments. The factors are grouped according to the themes above, with up to two quotations illustrating each factor. For each theme and factor, the total number of quotations, *N*, is given in parentheses. The total number of quotations overall is *N* = 462.

#### 1. Specific therapy components (total number of quotations = 204)

Numerous comments revealed that patients emphasize the impact of specific therapy components. Patients suggested a broad range of specific treatment approaches that are appraised as beneficial:

##### *Alternative therapies* (number of quotations = 122)

Many patients emphasized that a particular alternative therapy/therapist (e.g., Shiatsu, Yoga, Occupational Therapy) or a general alternative approach (e.g., Body Therapy, Creative Therapy, Relaxation Therapy) was associated for them with the experience of a ‘beneficial moment’. *Examples:* “Perceiving the body in a new way (e.g., Yoga, Shiatsu)”, “Getting to know relaxation techniques (Progressive muscle relaxation, Yoga, Shiatsu)”.

##### Mindfulness (meditation) (number of quotations = 38)

Respondents outlined that beneficial moments did occur when they payed attention to the present moment and the current experience. *Examples:* “To find mindfulness in everyday life”, “The fact that mindfulness permeates everything, coupled with mindfulness meditation, I have learned a great deal”.

##### Discussion-related group therapies/seminars (number of quotations = 28)

The patient’s participation in discussion-related group seminars as well as gaining knowledge about one’s own disease state through the communication during the seminar have been described as beneficial moments. *Examples:* “The *Quality of Life and Burnout Seminars* where the knowledge and context of my illness was explained to me”, “*Depression Seminar*: I felt well and understood”.

##### Medications (number of quotations = 12)

Patients also acknowledged that the pharmacological therapy that has been provided by the medical specialist is an important component of the therapeutic process. Aspects like the establishment, modification, or discontinuation of psychotropic drugs or other medications (e.g., painkillers) were mentioned. *Examples:* “[…] that I managed to utilize the positive effects of lithium”, “Drugs despite initial negative attitude”.

##### Exercise (number of quotations = 4)

Participating in sports therapies (i.e., Nordic Walking) of the psychiatric clinic were highlighted as a crucial therapy component.

*Examples*: “The sports offer of the psychiatric clinic gave me great pleasure and motivated me to schedule some time for sports in the future”; “The group *sports and game* was much fun and a welcome distraction.”

#### 2. Positive relationships (total number of quotations = 140)

Another dominant view was that the experience of a positive relationship forms the basis of a beneficial moment. Notably, the majority of responses highlighted that a good relationship with fellow patients is a central element during the stay at the clinic:

##### Relationship with fellow patients (number of quotations = 58)

Several patients highlighted that the establishment of relationships with one or more fellow patients was indispensable for trust building and a ‘beneficial moment’ in itself. *Examples:* “Fellow patients: Reflection of behaviour and unvarnished, but respectful feedback among each other”, “The exchange with other fellow patients, the deep relationships during this time”.

##### Relationship with the medical specialists (number of quotations = 36)

Furthermore, respondents emphasized the importance of establishing a relationship with the medical specialists. Beyond the experience of being understood, they also found the conversations with the medical specialists helpful for information transfer. *Examples:* “Consultations with medical specialists”, “The medical specialist has accompanied and assisted me. Furthermore, he believed that I will make it”.

##### Relationship with the nursing staff (number of quotations = 30)

Finally, some patients highlighted that the nursing staff enabled them to establish a trust-based relationship. Besides conversations, patients also mentioned the impact of nonverbal interactions with the nursing staff. *Examples*: “Relationship, trust, understanding of […] the nursing staff”, “Consultation with the nursing staff: The professional’s support was outstanding. She [the nurse] challenged me regularly with her targeted questions and specific hints; the contact with her influenced the therapeutic success substantially”.

##### Authentic relationship/feeling of belongingness (number of quotations = 12)

Some patients did appreciate the genuine experience of an authentic relationship and the feeling of belongingness. Patients described the importance of openness and authenticity in relationships as well as the consequent feeling of a sense of belonging to a more superordinate social context. In contrast to the categories above, these patients did not refer to a specific person (e.g., nursing staff, medical specialist, fellow patients). *Examples:* “The feeling of getting help and being saved when needed”, “The trust that I could have already gained after a few days. […] this trust has made everything possible: Openness, to allow being helped, hope, patience, new recognition and insights”.

##### Conversation with relatives (number of quotations = 2)

Two respondents mentioned that the conversation with their relatives under the guidance of the medical specialist was a ‘beneficial moment’ during their stay at the clinic. *Examples:* “Conversation with relatives”, “The conversation with the medical specialist and my partner: we found a good solution for the topic holidays at home”.

##### Professional skills/expertise (number of quotations = 2)

Notably, two patients appreciated that the professionals had a substantial expertise in their field. Patients emphasized that this provided the basis to resolve their problems. In this category, the expertise was named in the context of the therapeutic relationship. *Examples*: “Getting to know both medical specialists as highly competent”, “Competent professionals at all levels”.

#### 3. The setting and the environment of the clinic (total number of quotations = 52)

Implications of the setting and the environment of the clinic for patient care received considerable attention, and a mixture of opinions were offered about potential places for the experience of beneficial moments:

##### Nature/location (number of quotations = 28)

Interestingly, a considerable number of patients mentioned that the landscape of the clinic was a beneficial moment for them. More generally, some also mentioned that the atmosphere of nature (i.e., flora and fauna) around the clinic area was unique and healing for them. *Examples:* “Feel-good factor: Location of the clinic”, “Beneficial moments in nature as well as with the beautiful animals everywhere around the clinic (e.g., dogs, geese, horses, goats)”.

##### Tranquillity (number of quotations = 12)

Respondents acknowledged that the tranquillity as well as the silence of the clinical area was crucial for their own healing process. *Examples:* “Retreat to this quiet, beautifully situated place”, “In fact only the absolute peace”.

##### Service/hospitality (number of quotations = 8)

A few patients mentioned that the service and the hospitality of the clinic staff (i.e., kitchen, restaurant service, housekeeping and cleaning service) were important aspects during their stay. *Examples:* “The very attentive and helpful house cleaning team”, “The delicious food, the extremely friendly and fast service”.

##### Premises/architecture (number of quotations = 4)

The aesthetic style and ambience of the psychiatric clinic as well the area was highlighted by four patients to be an important part of their treatment success. *Examples:* “The opportunity to retire into a beautiful room, […], the aesthetics of the architecture and the furniture, the pleasant collective lounges (e.g., library)”, “The retreat: The cosy, spacious room with high ceilings, pleasant colours and the big bed”.

#### 4. Inpatients’ new insights (total number of quotations = 36)

A number of respondents commented on the association between new insights and the experience of beneficial moments; for example:

##### Functional subjective model of illness (number of quotations = 22)

Interestingly, some patients stressed that it was healing for them to find a functional explanation for their illness. Their original explanation changed through therapy into a functional explanation. *Examples:* **“**Understanding the cause of illness with the help of consultations with medical specialists and caregivers”, “Understanding the triggers and consequences of the depression and uncertainty”.

##### Gained insight through self-reflection (number of quotations = 10)

Ten respondents highlighted that they were able to self-reflect their situation with the help of the therapy and that this was a crucial aspect for their individual healing process. They stated to perceive thoughts and feelings more consciously. *Examples:* “To get an external view of the situation and my life”, “To perceive myself as me and to be perceived as such, not as a role of ‘wife’, ‘pastor’s wife’, or ‘mother’”.

##### Remoralization (number of quotations = 2)

Two patients acknowledged that they experienced a reduction in demoralization in the first phase of the therapy. They started to believe in their own problem-solving abilities. Furthermore, the therapy helped them to acknowledge that external and troubling factors are changeable. *Examples:* “Self-help and self-therapy”, “Finding again my *joie de vivre* (i.e., zest for life) after approximately 1.5 weeks after intake”.

##### Increase in expectation (number of quotations = 2)

Two respondents also stated that the therapy helped them to expect that the symptoms will decrease and will be manageable. They described a general increase in their quality of life and/or an increased resilience to recurrences. *Examples:* “Feeling ready to go home.”, “I am looking forward to being at home and expect being able to transfer all that I have learned into my daily life”.

#### 5. Factors unrelated to either therapy or the clinic (total number of quotations = 30)

Some comments suggested that beneficial moments take place outside the therapeutic context itself; for example:

##### Spirituality/pastoral care (number of quotations = 24)

The two pastors working in the clinic are employed and paid by the Protestant and Catholic churches respectively, and are not part of the therapeutic services of the clinic. A number of patients emphasized that the spiritual experience, the contact with the pastor of the clinic, or an inner experience triggered by spirituality or religiosity were important aspects of their healing process. *Examples:* “*Room of Tranquillity*, encounters with God”, “[…] and knowing to be supported in it by God, because he loves me and wants me to love myself”.

##### Exercise outside the therapy schedule (number of quotations = 6)

Some patients stressed that some type of sport or physical movement was associated with a beneficial moment for them. In all cases, the patients practiced sports outside the therapy schedule. *Examples:* “Regular jogging every day, linked with a daily sense of achievement and new confidence in one’s will”, “Sport is always healing for me”.

## Discussion

We set out to explore inpatients’ experience of beneficial moments during their clinical stay via a questionnaire. Furthermore, we intended to assess the distribution of specific, common and extra-therapeutic factors related to inpatients’ experience of beneficial moments. To the best of our knowledge, this is the first study focusing on events which patients themselves appraise as healing while classifying them as either specific, common, or extra-therapeutic.

To date, psychotherapy research is often divided into two kinds of models – the medical model that stresses the necessity of specific factors in the treatment procedure, and the contextual model, which defines psychotherapy as a therapeutic process [28]. In our study, we found that inpatients judge both specific and common factors as beneficial moments. Specific therapy components and expertise such as medications, group therapies and alternative treatments had a comparable number of quotations (*N* = 204) like common factors such as positive relationships, inpatients’ new insights, as well as the setting and environment of the clinic (*N* = 228). Extra-therapeutic factors had almost a negligible association with beneficial moments during inpatients’ stay at the psychiatric clinic (*N* = 30). On the one hand, specific factors such as therapy components and expertise were commonly mentioned by inpatients during their clinical stay. Notably, alternative therapies such as Shiatsu, Yoga, and Qi-Gong were by far the most frequently used content categories. This is in line with recent meta-analyses in various mental disorders, indicating that alternative approaches have a significant treatment effect, especially when compared to waiting lists or treatment-as-usual controls [35, 36]. On the other hand, common factors such as the relationship with the clinical staff, the feeling of belongingness, one’s own attitudes and the process of change through new insights were similarly often perceived as ‘beneficial moments’. It should be noted that the psychiatric clinic explicitly applies the Contextual Model as their treatment concept and that the multitude of quotations associated with common factors could partially be a result of this. Interestingly, inpatients put a greater emphasis on the relationship with fellow patients than on the relationship with the nursing staff or the medical specialists. Accordingly, research indicates that peer support for various mental disorders show significant effects on psychological wellbeing [37, 38].

**Fig. 2:**
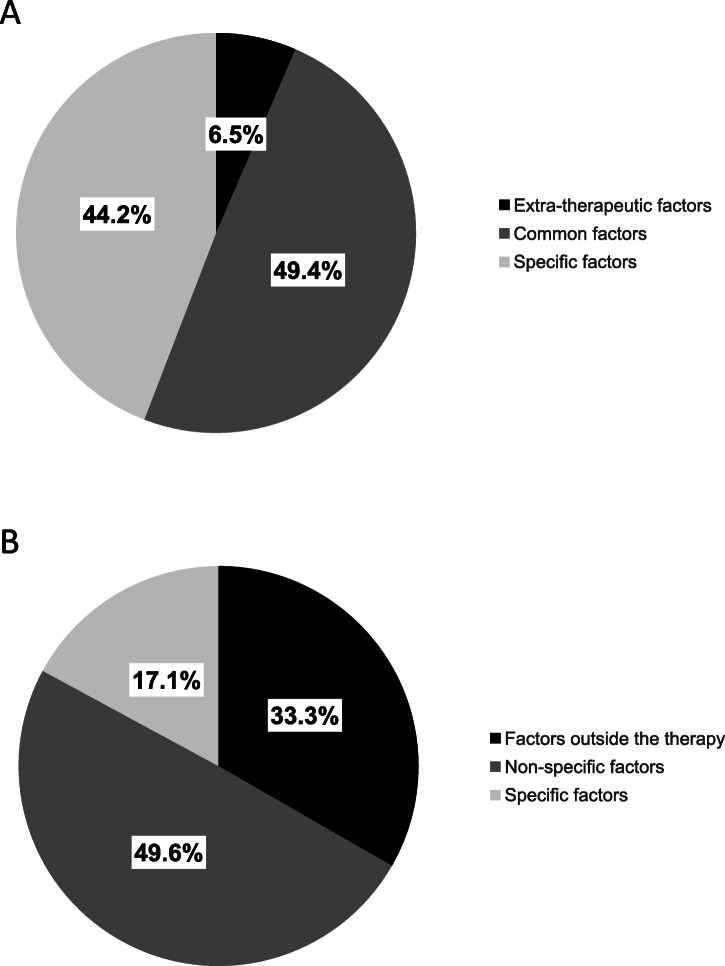
**a** Ranking of how important different factors are in treatment: Inpatients’ perspectives (*N* = 107) Note. Based on the main themes of the questionnaire. **b** Ranking of how important different factors are in treatment: Meta-analytic research findings in out- and inpatients (*N* = 2′805). Note. Factors outside the therapy = extra-therapeutic factors; non-specific factors = common factors. Reprinted from “The efficacy of non-directive supportive therapy for adult depression: a meta-analysis.” Clinical psychology review, 32 (4), 280–291, Copyright (2012), with permission from Elsevier

Furthermore, the results from our questionnaire can be compared with meta-analytical research findings [13] regarding the significance of specific, common and extra-therapeutic factors on symptom improvement (see Fig. 2a and b). Inpatients seem to weight the impact of specific factors higher than research, where only 17.1% of improvement is associated with specific factors [13]. Here, different kinds of explanations arise. First, patients could indeed experience the association between specific factors and beneficial moments as highly significant, especially in the clinical setting where the influence of extra-therapeutic factors is negligible. Notably, a stay at the clinic is indispensably linked with specific therapies. Also, in the referred meta-analysis [13], only five out of 31 studies were recruited from clinical samples. Second, one could argue that there is an increased focus on specific factors, emerging from the social as well as cultural context of our society where the medical model is prevailing. Third, it could be an artefact of patients’ cognitive dissonance: they decided to stay at the clinic and to engage in treatment. Hence, it is cognitively consistent to underestimate the influence of extra-therapeutic factors and attend more to the specific factors. Fourth, reinforcing common factors in the therapy process (e.g., through a good relationship between the medical specialist and the patient) could provide a basis for strengthening the potency of specific factors. Or, specific techniques and methods may enhance the feeling of being cared for. This would be in line with recent research findings indicating that participants’ positive expectations are most strengthened when a provider is *both* competent and empathic [39]. Hence, specific and common factors could influence and mutually reinforce each other according to inpatients experiences. For example, a quotation from the factor *Gained insight through self-reflection* states that “In several therapies, there were repeatedly moments when I was in balance, where I felt centred. I felt myself at the moment and it was good that way”.

The finding that there are marked differences between patients and researchers/practitioners in the ranking of how important different factors are in the inpatient care is not new. For example, Dowds and Fontana [40] examined patients’ and therapists’ satisfactions and disappointments in an inpatient psychiatric service. The authors differentiated between ‘primary interventions’ (i.e., specific therapy component such as group therapy, psychotherapy and medications), ‘adjunctive interventions’ (i.e., specific therapy components such as occupational therapy and nursing), as well as ‘hospital as retreat’ (i.e., common therapy components such as the setting and environment of the clinic as well as ‘informal relations’). Interestingly, patients rated all modalities as significantly more helpful than therapists. The authors argued that a likely explanation for the differences can be found in the expectations of the raters and that “patients come to the hospital in distress; they want to believe that help is there” (p.299). Similarly, in the context of nurse caring, a study indicated that patients’ and nursing staff’s perceptions of the most and least important nurse caring behaviours differed substantially; the authors concluded that “it is time to listen to the consumers’ views and perspectives” [41].

### Limitations

We would like to acknowledge some limitations of this study. First, there was a selected enrolment into the study as only inpatients with affective and adjustment disorders were analysed. It might be that different results would be found in different settings with other diagnoses. For example, patients with anxiety and depressive disorders differ in terms of their treatment goals [42]. Second, we did not control for the impact of additional patient characteristics such as their psychopathological history on the experience of beneficial moments. Third, patients acknowledged the setting and the environment of the clinic. It should be noted that the environment is most likely more favourable than that of many other inpatient units. Nevertheless, the results show that patients value their environments, and environments can be regarded as healing. Fourth, the investigated patients were treated in a private psychiatric clinic, offering a wide array of different treatment modalities. Generally, health insurance companies only cover a limited inpatient treatment for patients with adjustment disorders and depressive disorders. Thus, the generalizability of the presented results is limited. However, the investigated patients reveal suffering scores on healthcare questionnaires that are similar compared to those from patients treated in other Swiss inpatient units. Fifth, we did not ask patients who did not participate for reasons of not participating. This might have provided us with additional insights. Sixth, patients might have been primed in their answers by the questions earlier in the questionnaire. However, these questions covered the full range of patient experiences. Finally, the applied questionnaire has not been validated. However, similar terms like ‘beneficial moments’ have been used in psychotherapy research before (e.g., [30, 31]).

## Conclusions

Several conclusions can be drawn. First, our findings indicate that a good treatment in clinical practice should acknowledge that patients have their own definition of what is important in a therapeutic process and, moreover, that their perspectives might differ from research findings. Second, from an ethical point of view, inpatients should be informed about research findings which indicate that common factors are *more* important than specific factors in therapy improvement and that extra-therapeutic factors matter as well. Also, we recommend that practitioners should be open about their own personal expectations with the given treatment and ask about the inpatients’ idiosyncratic perspectives. This will increase the shared understanding of factors contributing to treatment improvement as well as the treatments’ credibility and plausibility. Third, for the clinical context, it could be beneficial to reinforce the interplay between specific techniques and common factors. For example, an emotionally warm and empathic style and a good relationship could be obtained *during* the specific treatment or *through* a specific method by that inpatients have the feeling of being understood and cared for. Fourth, qualitative investigations should be regularly consulted in clinical practice. Finally, future studies should focus on outpatients, where factors outside the therapy might have a greater impact on perceived beneficial moments.

### Outlook

Future studies should assess beneficial moments also six or 12 months after discharge to explore the retrospective and long-term sense of beneficial moments. Our results are drawn from a specific clinic with a focus on psychotherapy and an intense multimodal treatment; therefore, studies in other psychiatric clinics could enrich our understanding of the effects of therapeutic environment on inpatients’ subjective experience on beneficial moments. Furthermore, there has been an increased interest on the use of patient characteristics in order to match patients to the treatments that might be most suitable to them [43, 44]. Here, future studies might take qualitative approaches into account in order to gain a better understanding of inpatients’ perspectives and experiences.

## Data Availability

The dataset used during the current study are available from the corresponding author on reasonable request.

## Abbreviations

CBT: Cognitive-behavioural therapy
MBSR: Mindfulness-Based Stress Reduction
